# Enhanced Properties of Biodegradable Poly(Propylene Carbonate)/Polyvinyl Formal Blends by Melting Compounding

**DOI:** 10.3390/polym10070771

**Published:** 2018-07-13

**Authors:** Dongmei Han, Zhen Guo, Shou Chen, Min Xiao, Xiaohua Peng, Shuanjin Wang, Yuezhong Meng

**Affiliations:** 1 The Key Laboratory of Low-carbon Chemistry & Energy Conservation of Guangdong Province/State Key Laboratory of Optoelectronic Materials and Technologies, Sun Yat-Sen University, Guangzhou 510275, China; handongm@mail.sysu.edu.cn (D.H.); juice126126@163.com (Z.G.); stsxm@mail.sysu.edu.cn (M.X.); 2 School of Chemical Engineering and Technology, Sun Yat-Sen University, Guangzhou 510275, China; 3 Shenzhen Beauty Star Co., Ltd., Shenzhen 518112, China; chens@beautystar.cn (S.C.); alice@beautystar.cn (X.P.)

**Keywords:** poly(propylene carbonate), poly(vinyl formal), poly(vinyl alcohol), blends

## Abstract

Polyvinyl formal (PVF) was first synthesized via the reaction of poly(vinyl alcohol) (PVA) and formaldehyde. The synthesized PVF exhibits a high decomposition temperature, glass transition temperature, and low melting point compared to pristine PVA. The synthesized PVF can be melt processed at temperatures much lower than PVA. Poly(propylene carbonate) (PPC) and the as-made PVF were melt blended in a Haake mixer. The mechanical properties, thermal behaviors, and morphologies of PPC/PVF blends were investigated. Compared to the pure PPC, PPC/PVF blends show higher tensile strength and Vicat softening temperature. Thermogravimetric (TGA) result reveals that the thermal stabilities of PPC/PVF blends decreased with the increase of the content of PVF. Scanning electron microscopy (SEM) observation indicates that the interfacial compatibility of the PVF and PPC matrix is better than that of the PVA and PPC matrix. The PPC/PVF blends show much better comprehensive properties compared to pure commercial PPC, which provides a practical way to extend the application of PPC copolymer.

## 1. Introduction

The development of the global economy and increasing human activities are leading to historically unprecedented pressure on the earth, such as white pollution, climate warming, and energy crises. All of these issues are believed to be caused mostly by the mass use of petroleum-based plastic and the heavy release of carbon dioxide (CO_2_) into the atmosphere. Therefore, the reduction of massive CO_2_ emission and the research of degradable polymers have attracted considerable attention from scientists worldwide. In consideration of economy and ecology, it arises out of both scientific and practical interests to fix CO_2_ into degradable polymers with tailored properties. What is most heartening is that the copolymerization of propylene oxide (PO) with CO_2_ catalyzed by all kinds of catalysts has been widely reported and significant progress has been made [1,2,3,4,5,6,7,8,9]. 

**Scheme 1:**
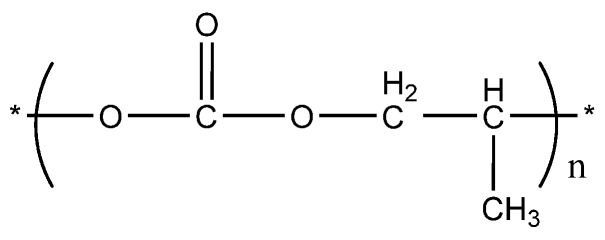
Structure of poly(propylene carbonate) (PPC).

More recently, an obvious breakthrough was achieved in the commercial production of poly(propylene carbonate) (PPC) from propylene oxide and CO_2_. PPC with a high molecular weight was synthesized in high yield using a supported zinc glutarate catalyst [10,11]. The structure of the resulting PPC is illustrated in Scheme 1. The as-made PPC, with an almost completely alternating molecular structure, exhibits not only high biodegradability in surroundings of both soil and buffer solutions but also perfect gas barrier properties [12]. The utilization of biodegradable PPC can not only fix carbon dioxide effectively for reducing greenhouse effect but also alleviate white pollution. Further, it also can do away with our dependence on petroleum to some extent. However, the relatively lower Vicat softening temperature and poor mechanical properties resulting from its ester molecular structure restrict its extensive application. For this reason, modification through melt blending with a corresponding polymer, which is seen as an economical and effective process, is imperative to further enhance its comprehensive performance and potential application.

In this regard, the blends of PPC with fillers, such as SiO_2_, CaCO_3_, montmorillonite, starch, fiber, and vermiculite, have been reported. However, because of the agglomeration of the fillers during blending, these blends displayed poor fluidity and dispersity. In this respect, blends with other polymers, such as ethylene-vinyl acetate copolymer (EVA), Ethylene vinyl alcohol copolymer (EVOH), polylactic acid (PLA), and polylactic acid (PBS), have been studied. Nevertheless, the properties of these blends were just improved to a very limited content and, especially, the low Vicat softening temperature of the blends restricted their application [13,14,15,16,17].

As a biologically friendly polymer, poly(vinyl alcohol) (PVA) has been widely used in various fields due to its outstanding film forming, adhesive properties, high tensile strength, and flexibility. However, its melt processing performance is very poor because its melt temperature (*T*_m_) is too close to its decomposition temperature (*T*_d_). Therefore, in order to solve this problem, various modifications of PVA have been reported, including physical and chemical methods [18,19,20,21,22,23]. Nevertheless, the plasticized PVA cannot blend with other polymers via the melting process because of the volatility of the small molecular plasticizing agent, which will have a detrimental effect on the materials’ properties.

For the aforementioned reasons, to successfully blend PPC with PVA through the melting processing, the plasticizing of PVA is a crucial step to decrease the melt processing temperature of PVA. In this work, we prepared polyvinyl formal (PVF) by the condensation reaction of formaldehyde with PVA in the presence of hydrochloric acid as a catalyst. The acetalization of formaldehyde with adjacent hydroxyl groups on PVA reduced the intra- and intermolecular hydrogen bonds among PVA, which subsequently endowed it with improved melt processability.

Finally, we obtained PVF with superior mechanical and thermal properties by means of acetal reaction. Then, PPC was modified by melt blending with PVF. On account of the interaction between the hydroxyl groups of PVF and the carbonyl groups of PPC, and the excellent mechanical and high Vicat softening temperature of PVF, the obtained PVF/PPC blends exhibit a high Vicat softening temperature and mechanical practical properties for practical application.

## 2. Materials and Methods

### 2.1. Materials

The PPC used in this work, with a number-average molecular weight of more than 250,000 and a polydispersity of 1.91, was kindly provided by Henan Tianguan (Enterprise) Group Co. Ltd., Nayang, China. PVA-205, with a degree of polymerization of 500 and a hydrolysis of 86.5%–89.0%, was purchased from Kuraray Tokyo, Japan. Aqueous formaldehyde (37%–40% formalin) (AR), hydrochloric acid (36%–38% HCL) (AR), and sodium hydroxide (AR) from Guangzhou Chemical Reagent Factory, Guangzhou, China were used without further purification. Both PPC and PVA were dried under vacuum at 80 °C for 24 h before using.

### 2.2. Preparation of PVF

**Scheme 2:**
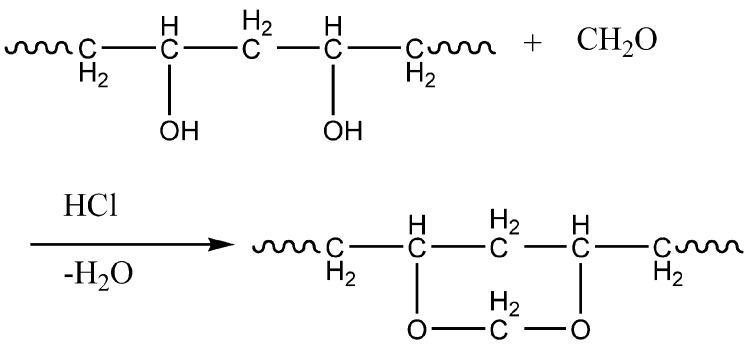
Synthesis scheme of poly(vinyl formal) (PVF).

PVF were synthesized from PVA and formaldehyde according to the literatures (Scheme 2) [24,25]. Prescribed amounts of PVA, formaldehyde, hydrochloric acid, and deionized water, for example, 100 g PVA, 100 mL deionized water, were charged into a beaker equipped with an overhead stirrer. The mixture was heated rapidly to 90 °C to dissolve PVA completely to obtain a clear PVA solution. Then, the solution was cooled to room temperature and mixed well with 100 mL of formaldehyde and 90 mL of hydrochloric acid. The reaction mixture was stirred at 60 °C for 4 h and the resulting product was cut into very small pieces. After neutralizing the acid with aqueous NaOH, the sample was dried in an air-circulating oven at 80 °C for 36 h.

### 2.3. Preparation of PPC/PVF Blends

The distinction of the melt flow rate (MFR) between PPC and PVF is vastly different. Therefore, the preparation of PVF/PPC blends has to be accomplished in two steps. First, PPC/70PVF blend with 70% PVF was prepared in a Haake Pheomix 600 mixer at 200 °C with a rotary speed of 60 rpm for 7 min. The chamber volume was 60 cm^3^ and 55 g of material was fed into the batch to fabricate the master batch of PVF. The resultant product was cut into very small pieces before being mixed with PPC. Then, the final binary blends of PPC/PVF were prepared by the melting compounding method from neat PPC and previous PPC/70PVF with different ratios. The contents of PVF in the final PPC/PVF blends were controlled at 30%, 40%, and 50%. The designation of PPC/30PVF indicates formulation with 30% PVF in weight. For easier comparison, the blends of neat PPC and PVA were also prepared under similar processing conditions in the mixer except that the mixing temperature was 185 °C. All the samples were enclosed in a tightly sealed desiccator to prevent moisture absorption.

### 2.4. Charaterization Techniques

Static tensile properties were measured at 25 °C and relative humidity (RH) of 50% using a SANS CMT tensile tester (Shenzhen, China), according to the standard of ASTM D638. The crosshead speed was set at 50 mm/min. Five specimens of each sample were tested and the average results were reported. All the samples were treated for 24 h at 25 °C and 50% RH before testing.

Vicat softening temperatures of specimens were measured at 50 °C/h and 10 N loads using a SANS HDT&VICAT Testing machine (Model: Zwk-1302-A, Shenzhen, China) according to the standard of ISO306.

Infrared spectra of the samples were recorded with a Perkin-Elmer type FTIR-100 spectrometer (Perkin-Elmer, Waltham, MA, USA) at a resolution of 4 cm^−1^ and a frequency range of 400–4000 cm^−1^. Typically, 16 scans are signal-averaged to reduce spectral noise. Specimens were casted to give thin transparent film around 20 μm thick tested by the transmission method.

DSC were measured with Netzsch 204 (Burlington, Germany) apparatus at a heating rate of 10 °C/min and in a nitrogen atmosphere. The flow rate of the nitrogen purge was 20 mL/min. The first heating scan of neat PVA and PVF ranged from 25 to 220 °C, but those of neat PPC and PVF/PPC blends ranged from −20 to 160 °C. The criterion used for the determination of the *T*_g_ was the mid-point of the curve and all the temperatures were evaluated from the second heating run to eliminate the previous thermal history.

Thermal decomposition performances of the samples were conducted on a Perkin Elmer TGA-6 instrument (Perkin-Elmer, Waltham, MA, USA) under a nitrogen protective atmosphere. The temperature ranged from 35 to 550 °C with a heating rate of 10 °C/min. All the samples were dried in a vacuum oven at 80 °C for 24 h prior to testing.

A TG/IR system, which combined a Perkin Elmer TGA-6 analyzer with a Perkin-Elmer FTIR-100 spectrometer, was utilized to perform TG/IR analysis. Samples of about 20 mg were pyrolyzed in the TG analyzer, and the evolved gases were led to the Perkin-Elmer FTIR-100 spectrometer directly through a connected heated gas line to obtain three-dimensional FTIR spectra. The specimens were measured at a resolution of 2 cm^−1^ and frequency range of 500–4000 cm^−1^ for four scans. TG measurements were carried out in a nitrogen atmosphere in the temperature range from 30 to 300 °C, and the heating rate was 20 °C/min in the range of 30–200 °C, while it was 10 °C/min in the range of 200–300 °C. The samples were dried in a vacuum oven at 80 °C for 24 h prior to testing.

NMR spectra were performed for ^1^H NMR on a Bruker NMR instrument (model DRX 400MHz, Bruker, Billerica, MA, USA) using dimethyl-d_6_ sulfoxide (DMSO-*d*_6_) as a solvent. The internal standard is tetramethylsilane, while the chemical shifts are given in ppm.

The samples were compressed to plates with a thickness of 1 mm and were fractured in liquid nitrogen for morphology observation with scanning electron microscopy (JEOL JSM-6380, Tokyo, Japan). Before the observation, the fractured surfaces were coated with a thin layer of gold.

## 3. Results

### 3.1. Molecular Structural Characterization of PVF

The reactions between PVA and formaldehyde in the presence of hydrochloric acid as a catalyst are depicted in Scheme [24,25].

The molecular structure of PVF was characterized by both FTIR and ^1^H-NMR techniques. The degree of acetalization (DA) was calculated from the results of ^1^H-NMR spectra. FTIR spectra of neat PVA and PVF are shown in Figure S1. The peaks at 3500–3400cm^−1^ are the –OH stretching vibration bands, which were weakened and shifted towards higher frequencies due to the cleavage of the intra- and intermolecular hydrogen bonding after acetalization. The peaks at 2935, 2859, 2784, and 2685 cm^−1^ correspond to –CH stretching bands. The bands at 1251, 1165, 1128, 1079, and 1016 cm^−1^ are ascribed to –C–O–C–O–C– stretching vibrations, which confirm the formation of a formal structure. Furthermore, the bands at 1721 and 1648 cm^−1^ seem to come from the –C=O groups of the residual acetate groups. These peaks are obviously weakened compared to those of PVA, resulting from the hydrolysis reaction of the residual acetate groups under alkaline conditions while neutralizing the acid using aqueous NaOH.

The ^1^H-NMR spectra of neat PVA and PVF are illustrated in Figure S2. All resonance peaks in the ranges 1.2–1.6 and 3.7–4.0 ppm correspond to methylene (–CH_2_–) and methine (–CH–) protons, respectively. The dioxymethylene (–O–CH_2_–O–) protons giving resonance in the region 4.6–5.0 ppm indicate the formation of the formal structure. The chemical shift assignments are in satisfactory accord with the work of Fujiwara [26,27]. The degree of acetalization is defined as the percentage of the reacted hydroxyl groups (DA). It can be calculated from the integrals of the NMR resonance. The peaks of protons on –O–CH_2_–O–(H2) represent the reacted hydroxyl groups, while the peaks of protons both on –CH(OH)–(H1) and –CH(OR)–(H3) refer to all the methine protons. Consequently, the DA calculated from H-NMR is about 67.6%.

### 3.2. Thermal Properties of PVF

Figure S3 presents the typical DSC thermogram curves of neat PVA and PVF. Due to the particular molecular architecture of PVF, the formal rings formed from the reaction between PVA and formaldehyde have an immobilization effect on the polymer chain. As a result, the glass transition temperature (*T*_g_) of PVF is higher than that of neat PVA. The higher *T*_g_ is expected to improve the thermal properties of PPC. In addition, there is no melting point of synthesized PVF because the formal rings can decrease the strong intra- and intermolecular hydrogen bonds within PVA chains and in turn destroy the regularity of the molecular structure. Consequently, it becomes an amorphous polymer which should be easily blended with amorphous PPC.

The thermal decomposition temperatures of the neat PVA and PVF are evaluated by the TGA technique, as shown in Figure S4. The decomposition temperatures of PVF in both the beginning and main degradation stages are higher than those of the pristine pure PVA. This improved thermal stability resulted from the acetalization of PVA with formaldehyde because formal rings exhibit better thermal stability compared to the hydroxyl groups on PVA chains, which restrain the elimination of hydroxyl groups during the thermal decomposition process. Compared with PVA, PVF exhibits a lower melting temperature, which in turn matches the melt blending temperature window of PPC.

### 3.3. Mechanical Properties of PPC/PVF Blends

**Figure 1:**
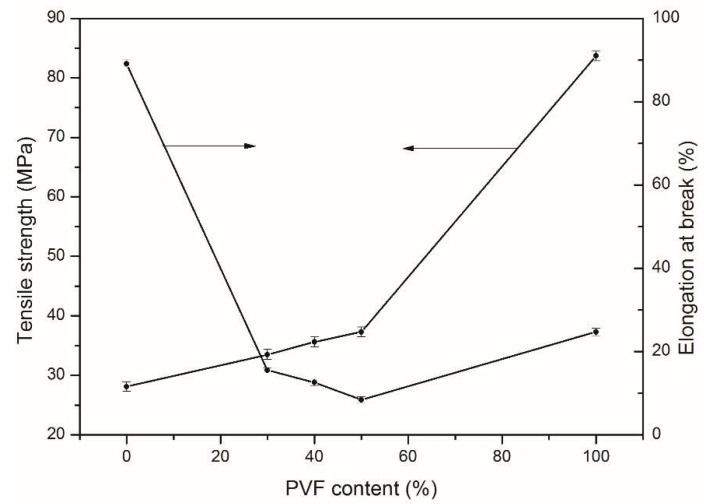
Tensile properties of PVF/PPC blends.

Figure 1 reveals the tensile strength and elongation at break of PPC/PVF blends. It can be seen that the tensile strength increases substantially and the elongation at break decreases sharply from 89% to 8.6% with increasing PVF content. This indicates that PPC/PVF blends become naturally brittle compared with the pristine PPC. This is believed to result from the relatively poor miscibility between PVF and PPC phases. Otherwise, after blending with PVF, the tensile strength of PPC/50PVF increases to 38 from 25 MPa of pure PPC. Considering the molecular structures of PPC and PVF, the enhanced reinforcement of the PVF/PPC blends are believed to arise from the hydrogen bond interactions between the carbonyl groups in PPC and the hydroxyl groups in PVF.

### 3.4. Thermal Properties of PPC/PVF Blends

**Figure 2:**
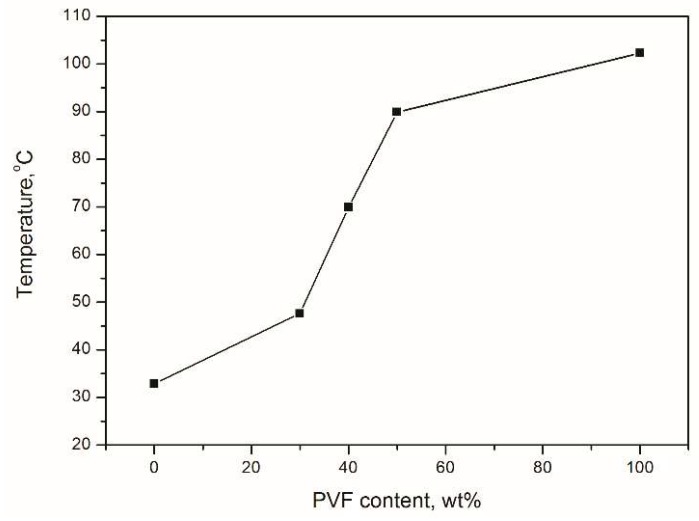
Vicat softening temperature of PPC/PVF blends.

The Vicat softening temperature (VST) is known to be one of the most crucial properties for the polymers used as plastic materials. The VST of pure PPC is about 34 °C, which is too low and limits the practical application of PPC. In this sense, the improvement in thermal properties for PPC is crucial to extend its practical application. Figure 2 shows the curve of VST values against the composition of PPC/PVF blends. It is apparent that a monotonic and nonuniform increasing of VST values is observed, implying the interaction between PVF and PPC [1,26]. The VST value shows a substantial increase of 115% when 40% of PVF is added. It is well known that the OH end groups of PPC in the melt state are easy to react with the cyclic formal. Though PPC and PVF are completely miscible, the reactive blending between the OH groups in the chain end of PPC with the cyclic formal in PVF still gives a slight cross-linking structure in the blends. These result in the improvement of Vicat properties and the tensile strength of the blends.

**Figure 3:**
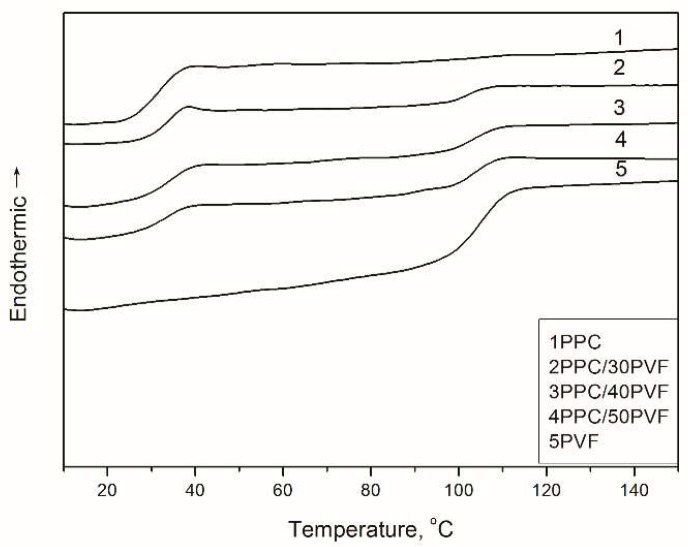
DSC traces of PPC/PVF blends.

Figure 3 presents the DSC traces of neat PPC, PVF, and PVF/PPC blends, and their glass transition temperatures (*T*_g_) are listed in Table 1. There is no melting temperature observed for all blends because of the amorphous nature of both PPC and PVF. With the increase of PVF content, the glass transition temperatures of PPC increase stably while the *T*_g_ of PVF lower steadily, demonstrating the interaction between PPC and PVF. The two glass transition temperatures of PPC and PVF shift closer towards each other than the pure PPC and PVF after blending, while the changes are very slight. The presence of the two independent *T*_g_ shows that the PVF and PPC are not completely miscible.

**Figure 4:**
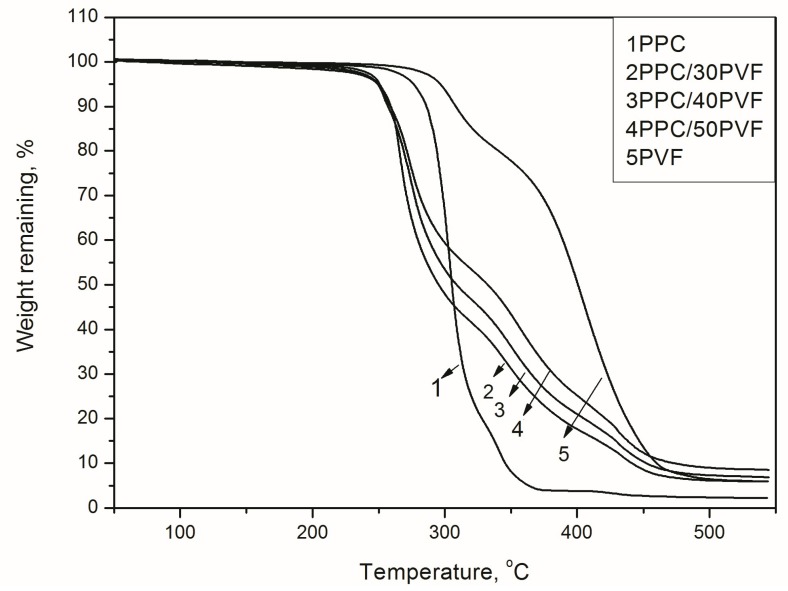
TGA curves for PPC, PVF, and PVF/PPC blends.

Figure 4 shows the thermogravimetric curves for neat PPC, PVF, and PVF/PPC blends. The detailed 5% weight loss temperature (*T*_-5%_), 10% weight loss temperature (*T*_-10%_), and maximum weight loss temperature (*T*_max_) are summarized in Table 1. It is evident that both *T*_max_ and *T*_-5%_ of PPC/PVF blends decrease with the increase of PVF content when compared to pure PPC. Notably, the pyrolysis of PPC is generated through two kinds of mechanisms: the unzipping reaction and chain scission. The unzipping reaction results from the terminal –OH groups forming cyclic propylene carbonate during thermal process [27]. Similarly, the hydroxyl groups of PVF may accelerate the unzipping reaction of PPC, leading to the decrease of weight loss temperature of PVF/PPC blends.

**Figure 5:**
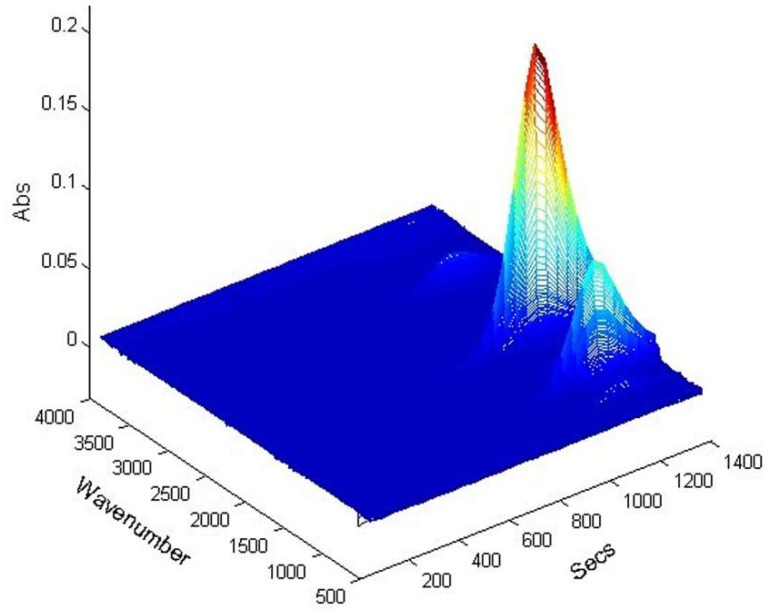
Three-dimensional FTIR spectra of 30PVF/70PPC.

In order to further confirm that the decline of the thermal stability of PVF/PPC blends, TG/IR was applied to follow the dynamic process of the PVF/PPC blend decomposition. The FTIR spectra in three-dimensions for the degradation dynamic process of PVF/PPC blends obtained from this technique is illustrated in Figure 5. The three-dimensional FTIR spectrum of pyrolysis confirms that the degradation reaction of PVF/PPC is normally the unzipping reaction of PPC at the testing temperature range from 100 to 300 °C. Only one pyrolysis product of cyclic propylene carbonate is detected, as shown at the peak of around 1800 cm^−1^. This result reveals that the hydroxyl groups on PVF initiate the unzipping reaction of PPC and results in the slight decrease of the thermal stability of PVF/PPC.

### 3.5. Microstructure of PVF/PPC Blends

**Figure 6:**
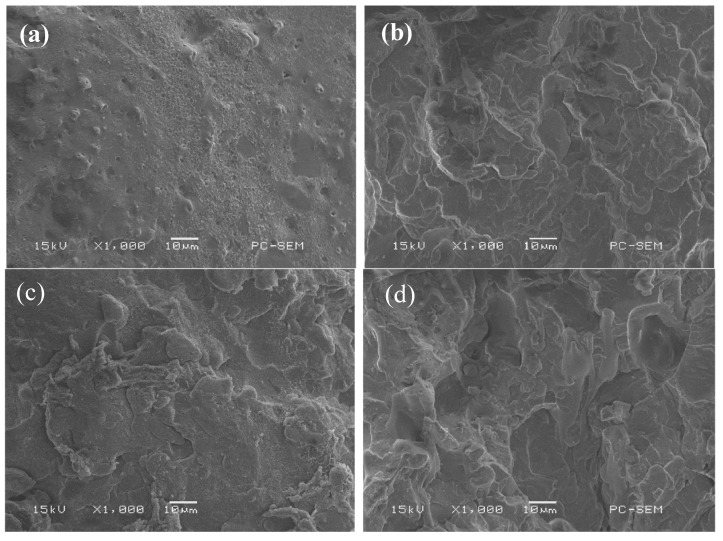
SEM micrographs of PVF/PPC blends. (**a**) PPC/30PVA; (**b**) PPC/30PVF; (**c**) PPC/40PVF; (**d**) PPC/50PVF.

Microstructure examination using scanning electron microscopy (SEM) is a direct way to determine the miscibility of the components of a blend. For the PPC/30PVA (Figure 6a) blend, the PVA phase seems to be dispersed in granular form within the PPC matrix. However, there are numerous voids observed resulting from nonmelted PVA. The PVA particles behave as inorganic-like particles; therefore, the mechanical properties of PVA/PPC are rather poor.

Figure 6b shows the morphology of the PPC/30PVF blend. The PVF phase is well melted and dispersed in the PPC matrix without noticeable gaps. This fact indicates that the interaction between PVF and PPC is improved compared to that between PVA and PPC, which in turn leads to better mechanical properties. With increasing the PVF content (Figure 6d), the two-phase microstructure still exists with some of smooth dots. These dots indicate the weak interfacial adhesion between two phases which then leads to poorer mechanical properties. As mentioned above, the OH end groups of PPC can react with the cyclic formal groups of PVF, which results in a cross-linked structure in the blends. The reactive blending improved the Vicat temperature of the PPC/PVF blends with the increase of PVF content.

## 4. Conclusions

PVF/PPC blends were prepared through a melting processing. Mechanical measurements showed that the tensile strength of PVF/PPC blends increases significantly with increasing PVF content compared to the neat PPC. Moreover, the thermal analysis revealed that the Vicat softening temperature increases by 115% with 40 wt % PVF addition. Finally, the morphological observation demonstrated that the PVF/PPC blends show an obvious two-phase microstructure. The interfacial adhesion between PVF and PPC is much better than that between PVA and PPC. The improved compatibility of PVF/PPC blends results in superior mechanical strength when compared with pure PPC.

## Figures and Tables

**Table 1:** Thermal properties of PPC, PVF, and PPC/PVF blends.

PPC/PVF	*T*_g_ (°C)		*T*_-5%_ (°C)	*T*_-10%_ (°C)	*T*_max_ (°C)
100/0	32.2	/ ^a^	257.9	275.3	284.0
70/30	33.6	102.5	250.2	259.1	267.1
60/40	34.2	103.9	249.4	257.0	275.7
50/50	34.4	104.3	249.3	258.7	273.6
0/100	/ ^a^	104.9	296.5	308.0	411.6

^a^ only one *T*_g_.

## Supplementary Materials

The following are available online at http://www.mdpi.com/2073-4360/10/7/771/s1.
