# Supplementary material for: Enhanced Properties of Biodegradable Poly(Propylene Carbonate)/Polyvinyl Formal Blends by Melting Compounding
Source: Polymers (Basel). 2018 Jul 13;10(7):771. doi: 10.3390/polym10070771 (PMC6403954; doi:10.3390/polym10070771)
Supplement: Supplementary file 1 [file polymers-10-00771-s001.pdf]

Enhanced Properties of Biodegradable Poly(propylene carbonate)/Polyvinyl Formal Blends by melting compounding

Dongmei Han[1, 2, +], Zhen Guo[1, +], Shou Chen[2], Min Xiao[1], Xiaohua Peng[2], Shuanjin Wang[1,*], YuezhongMeng[1*]

1 The Key Laboratory of Low-carbon Chemistry & Energy Conservation of Guangdong Province/ State Key Laboratory of Optoelectronic Materials and Technologies, Sun Yat-Sen University, Guangzhou 510275, PR China; handongm@mail.sysu.edu.cn (D.H.); juice126126@163.com (Z.G.); chens@beautystar.cn (S.C.); stsxm@mail.sysu.edu.cn(M.X.); alice@beautystar.cn (X.P); wangshj@mail.sysu.edu.cn (S.W.); mengyzh@mail.sysu.edu.cn (Y.M.)

2 School of Chemical Engineering and Technology, Sun Yat-Sen University, Guangzhou 510275, PR China

3 Shenzhen Beauty Star Co., Ltd, 518112 Shenzhen, China;

* Correspondence: mengyzh@mail.sysu.edu.cn (Y.M.); wangshj@mail.sysu.edu.cn (S. W.); Tel.: +86-20-84114113

+ These authors have contributed equally to this work

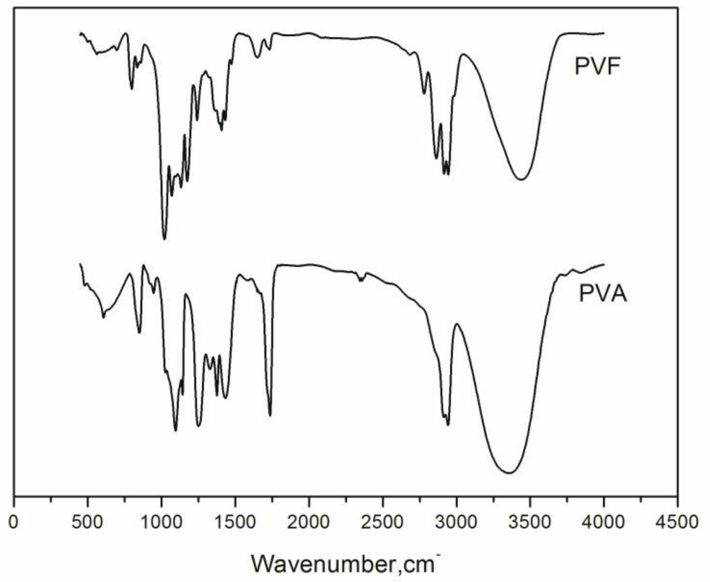

Fig S1 FTIR spectra of PVA and PVF

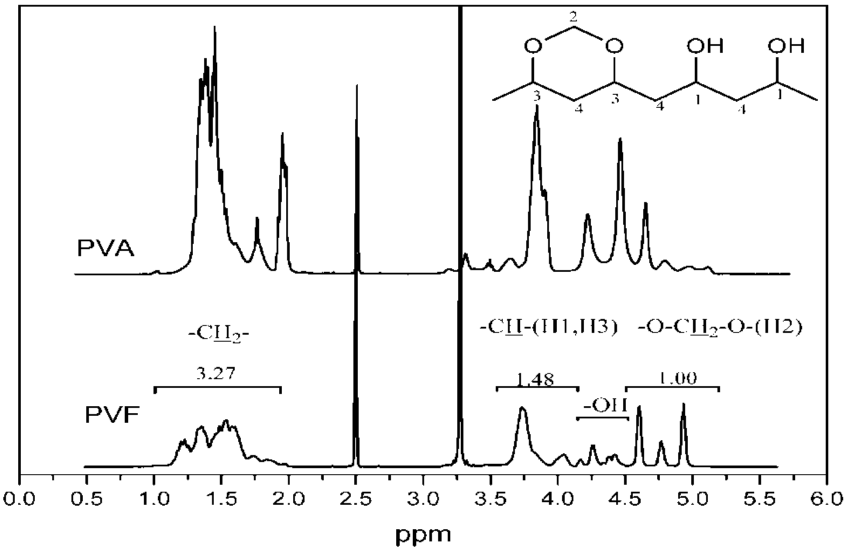

Fig S2 ^{1}H-NMR curves of PVA and PVF

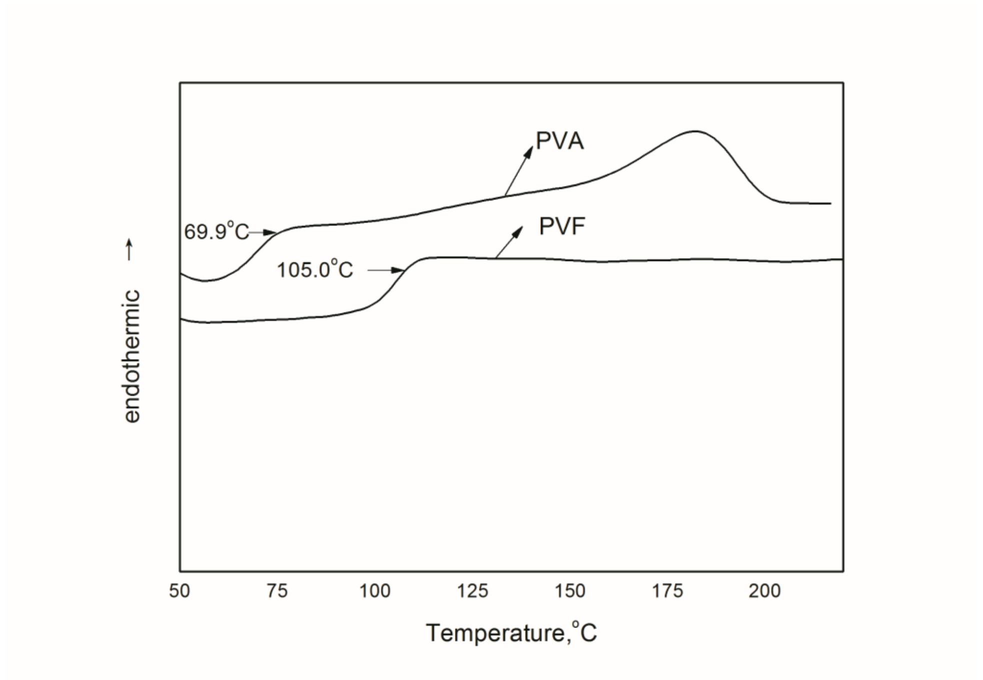

Fig. S3 DSC curves of PVA and PVF

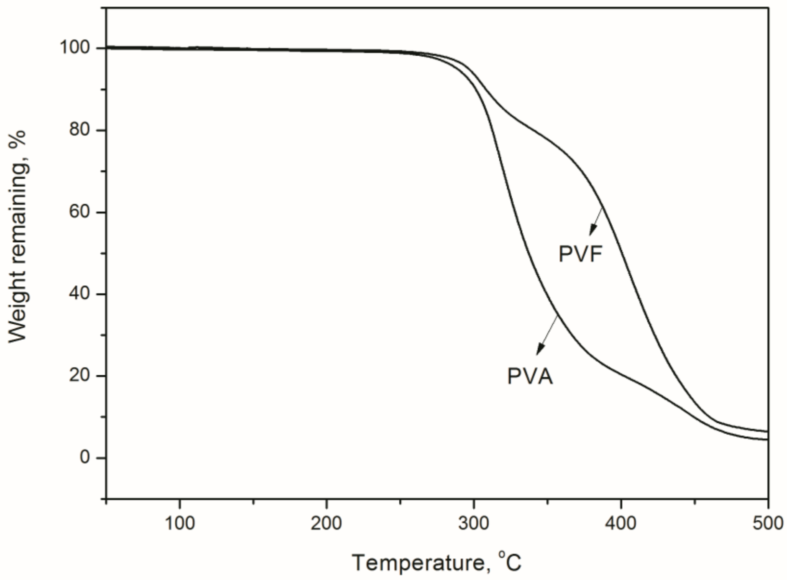

Fig. S4. TGA curves of PVA and PVF
